# Therapeutic Value of *Lactobacillus gasseri* 345A in Chronic Constipation

**DOI:** 10.1111/nmo.70012

**Published:** 2025-03-03

**Authors:** Stefan Roos, Atti‐La Dahlgren, Yu‐Kang Mao, Anton Pallin, Andrew M. Stanisz, Paul Forsythe, Wolfgang Kunze, Per M. Hellström

**Affiliations:** ^1^ Department of Molecular Sciences, Uppsala BioCenter Swedish University of Agricultural Sciences Uppsala Sweden; ^2^ BioGaia AB Stockholm Sweden; ^3^ Department of Medical Sciences Uppsala University Uppsala Sweden; ^4^ Brain‐Body Institute McMaster University Hamilton Ontario Canada; ^5^ Department of Psychiatry McMaster University Hamilton Ontario Canada

**Keywords:** constipation, intestinal microbiota, Lactobacillus, motility, probiotics

## Abstract

**Background:**

Chronic constipation is a prevalent, burdensome gastrointestinal disorder whose etiology and pathophysiology remain poorly understood. Differences in the composition of the intestinal microbiota have been shown between constipated patients and healthy people. Data indicate that these microbial differences contribute to the disorder.

**Methods:**

Preclinical studies in mice examined the effects of *Lactobacillus gasseri* on intestinal motility ex vivo, the reversal of motility inhibition by μ‐opioid receptor agonists ex vivo and in vivo in mice, and the effects on capsaicin‐stimulated transient receptor potential vanilloid 1 (TRPV1) in Jurkat cells. Thereafter, a clinical study of 40 women with functional constipation was conducted to investigate the effects of *Lactobacillus gasseri* with a randomized parallel design. After 14 days of baseline recording, treatment with *Lactobacillus gasseri* or placebo was given over 28 days, with 14 days of follow‐up. Outcomes with complete spontaneous bowel movements (CSBM), spontaneous bowel movements, emptying frequency, abdominal pain, time spent for defecation, Bristol stool form scale, use of rescue laxatives, and impact on sex life were investigated.

**Key Results:**

In preclinical studies, *Lactobacillus gasseri* increased intestinal motility in an ex vivo model, reversed the motility inhibition caused by μ‐opioid receptor agonist ex vivo and in vivo in mice, and counteracted capsaicin‐stimulated activity of TRPV1 in Jurkat cells. In the clinical trial, *Lactobacillus gasseri* showed a significant reduction in abdominal pain, along with a correlation and tendency for an increased number of CSBM. Few adverse events were encountered.

**Conclusions and Inferences:**

Treatment with *Lactobacillus gasseri* can alleviate pain sensations in functional constipation, possibly with an improved bowel‐emptying function.

## Graphical abstract

Preclinical studies show *Lactobacillus gasseri* to increase intestinal motility and counteract capsaicin‐stimulated transient receptor potential vanilloid 1. In a clinical trial, *Lactobacillus gasseri* reduced abdominal pain, along with a correlation for increased complete spontaneous bowel movements. Treatment with *Lactobacillus gasseri* can alleviate pain in functional constipation with improved bowel emptying function.

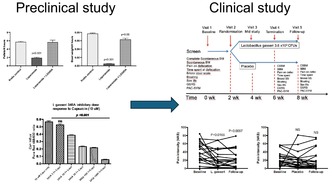

**Summary**

- *Lactobacillus gasseri* increases motility and dampens TRPV1‐mediated pain signaling.
- In humans, the bacterium may cause increased bowel movements and pain relief.

## 1 Introduction

To investigate the action of lactobacilli on the physiological functions of the gastrointestinal tract, we used a strain of the species *Lactobacillus gasseri* 345A for experimental animal studies ex vivo and in vivo, and cell cultures to study motility and pain sensation.

Constipation with abdominal distension and pain has an estimated prevalence of 20% and affects people of all ages and genders with an impact on their quality of life [1]. Primarily, women and adults over 60 years of age are affected [2, 3]. The etiology and pathophysiology of chronic constipation remain poorly understood, and the clinical management remains challenging. Growing evidence indicates that changes in the intestinal microbiota may contribute to constipation and related symptoms. Studies reveal variations in the composition and stability of the gut microbiota in constipated patients compared with healthy controls.

The effect of *L. gasseri* was studied on intestinal segments ex vivo under baseline motility conditions and after inhibition of motility by activation of μ‐opioid receptors. In addition, the effect of the bacterium was studied on a Jurkat cell line for inhibition of capsaicin‐mediated calcium signaling through the transient receptor potential vanilloid 1 (TRPV1), since this receptor is implicated in intestinal pain transmission and motility in pathological conditions [4, 5, 6].

In chronic constipation, the intestinal microbiota is characterized by a decrease in obligate bacteria (e.g., *Lactobacillus*, *Bifidobacterium*, and *Bacteroides* spp.) [7] and a parallel increase of pathogenic bacteria (e.g., *Pseudomonas aeruginosa* and *Campylobacter jejuni* ) [6, 8]. Using 16S rRNA gene pyrosequencing, a decreased abundance of *Prevotella* and increased *Firmicutes* spp. was found in constipated patients [9]. Furthermore, the butyrate‐producing *Coprococcus*, *Roseburia*, and *Faecalibacterium* tended to be increased in constipated patients [10, 11]. These studies indicate that constipation could be a consequence of intestinal dysbiosis, which is why the present study of *L. gasseri* in different experimental models and in a clinical study was undertaken.

The initial cell and animal experimental studies were carried out to obtain a translational background for a subsequent clinical study on constipation in humans. The aim of the clinical study was to evaluate the effects of oral administration of *L. gasseri* 345A over 28 days on the number of complete spontaneous bowel movements (CSBM), spontaneous bowel movement (SBM) frequency, abdominal pain and distension, time spent for defecation, stool consistency, use of supplementary laxatives, and sex life. Furthermore, the fecal microbiota was analysed for differences before and after intervention with the bacterium.

## 2 Methods

### 2.1 Bacterial Strain, Cultivation, and Chemicals

A Lactobacillus strain was identified by 16S rRNA gene sequencing (GenBank accession number MW221273), named *Lactobacillus gasseri* 345A. In the German Collection of Microorganisms and Cell Cultures, the strain is designated DSM 27123. The bacterium was donated by BioGaia AB (Stockholm, Sweden) and stored in 15% glycerol at −70°C. The strain was cultured at 37°C in Man‐Rogosa‐Sharpe (MRS) broth or on MRS agar plates (Difco Laboratories, Sparks, MD, USA; the latter under anaerobic conditions). Cell numbers were determined optically, and viability was checked by cultivation on MRS agar. The bacteria were stored frozen (−80°C), 1‐mL aliquots with 5 × 10^9^ cells in MRS broth. Cells from frozen stocks were thawed and centrifuged at 2000 rpm for 15 min, and the pellet was suspended in Krebs buffer. Then, the suspension was again centrifuged, and the cells were removed and resuspended in Krebs at the original concentration.

Capsaicin was obtained from Sigma‐Aldrich (Oakville, ON, Canada) and dissolved in ethanol to prepare stock solution aliquots. On the experiment day, aliquots were diluted in Krebs solution with final ethanol concentrations of 0.001%–0.01%. Loperamide was purchased from Sigma‐Aldrich and dissolved in saline with a concentration 1 mmol/L.

### 2.2 *Ex Vivo* Intestinal Motility Model

Adult male BALB/c mice (20–30 g) procured from Charles River Laboratories (Wilmington, MA, USA; http://www.criver.com) were used for the experiments. The mice were killed by cervical dislocation, adhering to Canadian Council on Animal Care guidelines and approved by the McMaster University Animal Research Ethics Board. Male mice were chosen to obtain meaningful results with a small sample size. By evading female mice' variable hormones, the background noise of the obtained data should be reduced, which may improve the outcome of results.

Since previous data in humans show small bowel motor abnormalities in chronic constipation [12, 13], experiments were carried out on excised small bowel. For motility experiments, a 4‐cm‐long jejunal segment was excised, mounted in a 20‐mL organ bath, and submerged with oxygenated Krebs solution with the composition (mM): 118 NaCl, 4.8 KCl, 25 NaHCO_3_, 1.0 NaH_2_PO_4_, 1.2 MgSO_4_, 11.1 glucose, and 2.5 CaCl_2_ bubbled with carbogen gas (95% O_2_ and 5% CO_2_). The Krebs solution was pre‐warmed to 34°C–35°C and superfused (2–4 mL/min) on the serosa of intact jejunal segments. In the motility experiments, the oral and anal ends were cannulated, and the lumen was perfused with carbogen‐gassed Krebs as described [14]. The intraluminal compartment was perfused with buffer at room temperature (19°C–22°C) at 0.5 mL/min. At the start of the experiments, the organ bath was perfused with pre‐warmed (34°C), carbogen‐gassed Krebs solution at 5 mL/min without *L. gasseri* 345A. Then, the head to the lumen inflow pressure was raised to 3 hPa, and recordings were done at this pressure level. Immediately before the exposure of the organ to *L. gasseri* 345A, bacterial cells were diluted to working concentrations of 10^9^ CFU with Krebs buffer. The luminal inflow was switched between Krebs solution with or without bacteria [15].

Phasic intraluminal pressure increases were measured at the midpoint of the longitudinal axis of the jejunal segment using an intraluminal, Krebs‐filled, 0.58‐mm external diameter polyethylene tube [15]. The polyethylene tube, emerging from the anal end, was attached to a COBE (Sorin Biomedical Inc., Irvine, CA, USA) pressure transducer. The voltage signal was amplified using a Grass LP 122 amplifier (Astro‐Med, Brossard, QC, Canada) and then digitized using a MiniDigi 1A A‐D converter and Axoscope 9 software (Molecular Devices, Toronto, ON, Canada). Signals were stored on a PC computer and analysed using Axon pCLAMP 9 software (Molecular Devices).

Video imaging of the contracting gut was done with a video camcorder (JVC Everio Hard Disk Camcorder Model GZ‐MG155U) placed 10 cm above an intestine. Recording was started in synchrony with the pressure recording using an 8–12 cm field view during each experiment. The raw camera output was 30 frames per second (fps), and 10‐min video clips were excised from the MOD file using Avidemux 2.5.0; (http://www.avidemux.org). The clips were converted into MOV format using Zune converter 1.1 (http://ffmpeg.mplayerhq.hu). Final video clips were resampled with a resolution 384 × 256 pixels and 25 fps.

Video recordings were analyzed using in‐house image processing software (StMap) developed as a plug‐in for NIH ImageJ (version 1.43c; NIH, Bethesda, MD, USA). The software converts images of the video into a black‐and‐white silhouette, a spatiotemporal map using an edge detection routine. The routine first measures the diameter at each position along the gut and then represents the physical diameter at each position as a black–white hue value (range 0–255). As the software reads through each 10‐min clip, a spatiotemporal map is generated with alternating bands of light and dark hues that contain three sets of information: position along the gut, timepoint, and gut diameter. Hence, the spatiotemporal map becomes a motility “fingerprint” whose sensitivity is critically important when defining detailed motility effects of specific bacterial strains [15].

The motility‐stimulating effect of *L. gasseri* was further investigated employing a μ‐receptor opioid agonist, 30 μmol/L loperamide added to the serosa, for the inhibition of the motility. The luminal inflow was switched between Krebs alone with no bacteria, Krebs with bacteria, and Krebs with bacteria and loperamide.

### 2.3 *In Vivo* Intestinal Motility Model

The effect of *L. gasseri* 345A was also studied in mice in an in vivo loperamide motility model. Before the experiments, animals were fasted for 12 h but had free access to water. During the experimental period, animals had free access to water. Loperamide, 7–10 mg/kg, was given by oral gavage to each mouse (*n* = 5). Then, 15 min later, a suspension of *L. gasseri* 345A (10^9^ CFU) was given orally, and fecal collection started another 15 min later, with repeat sampling at 60‐min intervals for 4 h. For comparison, another two bacterial strains, *L. reuteri* NN and *L. gasseri* NN, were used at the same dosage. The total number of fecal pellet output was measured, as well as the fecal wet weight over the 4‐h period. All procedures were conducted according to Canadian Council on Animal Care guidelines as approved by McMaster University Animal Research Ethics Board.

### 2.4 TRPV1 Pain Receptor Model

The effect of *L. gasseri* 345A on capsaicin activation of the TRPV1 pain receptor was studied in a Jurkat cell culture model. The human Jurkat cell line Clone E6‐1 (ATCC TIB‐152) was obtained from the American Type Culture Collection (Rockville, MD, USA). The cell line was authenticated by the ATCC Cell Authentication service using short tandem repeat profiling techniques (data not shown). Cells were maintained in Roswell Park Memorial Institute‐1640 medium supplemented with 2 mM glutamine, 100 units/mL penicillin, 50 μg/mL streptomycin, and 10% fetal bovine serum. Cells were passaged every 3–4 days, with experiments performed between passages 3–8 [16].

Jurkat cells in culture medium were seeded onto 22 mm diameter polylysine‐coated glass coverslips at a density of 50,000 cells per coverslip and cultured overnight. Medium was removed, and coverslips were washed with Tyrodes medium (mM: NaCl 145, KCl 2.5, HEPES 10, glucose 10, MgCl_2_ 1.2, CaCl_2_ 1.5, pH 7.4, supplemented with bovine serum albumin 1 mg/mL). Cells were incubated with Tyrodes medium containing Fluo‐3 AM 2 μM and Fura Red AM 10 μM in the dark for 30 min at 25°C, washed three times for the removal of extracellular dye, and incubated for another 30 min at 25°C. Cells were then imaged on a PerkinElmer Ultraview confocal microscope running UltraView 4.0 software. Fluorescent dyes were excited at 488 nm using an argon ion laser, and fluorescence emission was collected using 525/25 nm bandpass and 650 nm long‐pass filters for Fluo‐3 and Fura Red, with data expressed as the percentage change in Fluo‐3:Fura Red emission intensities in individual cells before and after treatment with capsaicin to activate the TRPV1 with *L. gasseri* at different concentrations.

### 2.5 Clinical Study

Forty women with functional constipation were studied in a 28‐day randomized, double‐blind, parallel group, placebo‐controlled explorative study with the objective of determining changes in defecation characteristics after oral supplementation with *L. gasseri* 345A. The study was approved by the Regional Uppsala Ethics Board 2015/276/4 (2016–10‐19) and registered on ClinicalTrials.gov (no.: NCT02592200).

Baseline data were collected along with the medical and surgical history, as well as the medications taken within 1 month before screening and during the study.

Patient inclusion was based on the following criteria: women, age 18–59 years, able to provide informed consent, functional constipation defined by the Rome III criteria [17] with symptoms for > 3 months and an onset > 6 months prior to diagnosis (for women aged 49–59 years, symptoms were required for ≥ 12 months), body mass index (BMI) 18–29 kg/m^2^, no use of other probiotics from the screening visit throughout the study period, adequate contraception (menopause > 1 year; or if childbearing potential highly efficient contraception), ability to understand and comply with the requirements of the study as judged by the investigator. Subjects with lactose intolerance and gluten‐related disorders were eligible to participate in the study while on a lactose‐free or gluten‐free diet. Exclusion criteria were hypersensitivity or allergy to the investigational product, to chemically related products, or placebo, organic cause of constipation (i.e., hypothyroidism, Hirschsprung's disease, cystic fibrosis); irritable bowel syndrome; anorectal pathology; previous resection of the small intestine or colon, or bariatric surgery; spinal anomalies or injuries; use of antibiotics or *L. gasseri* supplements within 2 weeks prior to screening; alarm symptoms (i.e., rectal bleeding, weight loss, jaundice); mental or behavioral disorders at the discretion of the investigator.

All subjects had a standard physical and clinical chemistry examination at screening and follow‐up visits. The physical examination included the cardiovascular system with systemic systolic and diastolic blood pressure, pulse rate, respiratory system, abdomen, skin, and nervous system. The following clinical chemistry parameters were measured: blood hemoglobin and leucocyte count, serum levels of alanine aminotransferase and aspartate aminotransferase, creatinine, thyroid‐stimulating hormone, alkaline phosphatase, transglutaminase IgA, fasting plasma glucose, HbA1c, albumin, calcium, and C‐reactive protein. A pregnancy test was performed before entering the study.

After recruitment and baseline recording over a 14‐day period, subjects were randomized to receive either *L. gasseri* or placebo; 20 subjects in each study arm with an intervention period of 28 days followed by a 14‐day follow‐up period. During the 28‐day study period, subjects were not allowed to use other probiotic products, antibiotics, laxatives, or enemas. As rescue medication, bisacodyl 10 mg suppositories were supplied after ≥ 5 days without a bowel movement. Subjects were not permitted to take any medications that could interfere with symptom evaluation or use other unlicensed investigational medicinal products or supplements. In addition, subjects were instructed not to change their daily dietary intake after inclusion in the study.

Treatments were given with the active investigational product *L. gasseri* 345A or placebo randomized at a 1:1 ratio. Slow‐release capsules composed of hypromellose, gellan gum, and titanium dioxide with maltodextrin and magnesium stearate (anti‐caking agent) with *L. gasseri* 345A (> 1 × 10^9^ CFU) (batch 5XINX40) or placebo (batch 5XINX61) were used. Participants were prompted to take one capsule BID with breakfast and dinner. Treatment compliance was monitored at the follow‐up by capsule accountability and number of ingested capsules per day as recorded in the study diary. Unblinding of the study was done after the database lock.

### 2.6 Efficacy and Safety

The efficacy variables of bowel habits were change in frequency of CSBM or SBM, pain sensation at each bowel movement according to a VAS 0–100 scale, time spent during each defecation, and stool consistency. CSBM was defined as a bowel movement that led to a feeling of complete passage of stool (rather than partial or incomplete evacuation), while SBM was defined as any bowel movement (both of which without any laxative within the previous 24 h). Stool consistency was evaluated according to the Bristol Stool Form scale at each bowel movement. Abdominal distension was assessed by measuring waist circumference. The impact on sex life was assessed by using a VAS 0–100 scale. All questionnaires were translated and validated for the Swedish language. The time and date of laxative use were recorded by the subjects in the study diary.

Adverse events (AEs) were monitored by physical examination and safety laboratory reports. The study was monitored on a regular basis to ensure that the study was conducted and documented properly. Monitoring confirmed that informed consent was obtained from all participants, AEs were reported and data accurately recorded, the investigational product was stored correctly, and drug accountability was continuously documented. The accuracy of study data reported in the eCRF was verified through the review of the source data by monitoring services.

### 2.7 Quantification of *Lactobacillus gasseri* in Feces Before and After Intervention With qPCR

#### 2.7.1 DNA Preparation

DNA was prepared from fecal samples by using the QIAamp Fast DNA Stool Mini Kit (Qiagen, Toronto, Ontario, Canada). Preparations were performed according to the instructions from the manufacturer, with few modifications. One hundred mg of fecal sample was weighed in a 2‐mL bead‐beating tube (0.25 mL of 0.1 mm zirconia/silica beads (BioSpec, Bartlesville, OK, USA) in a 2‐mL microtube PP) and placed on ice. InhibitEX 0.5 mL buffer was added to each stool sample, and a bead‐beating step was performed at speed 5.0 for 3 × 45 s (FastPrep‐24 Instrument, MP Biomedicals, Solon, OH, USA). Additional steps were performed according to the manufacturer's instructions.

#### 2.7.2 Standard DNA Preparation

Chromosomal DNA of *L. gasseri* 345A was isolated and used for the creation of a qPCR standard curve. DNeasy Blood & Tissue kit (Qiagen) was used according to the manufacturer's instructions with modifications. Cultured cells (1 mL) of *L. gasseri* were harvested by centrifugation for 10 min at 5000 ***g*** (7500 rpm). The bacterial pellet was resuspended in 180 μL enzymatic buffer and incubated for 60 min at 37°C. This was followed by a bead beating step using 0.25 mL of 0.1 mm Zirconia/silica beads in a 2 mL tube at speed 5.0 for 3 × 45 s (FastPrep‐24 Instrument, MP Biomedicals). The bead beating samples were incubated for 30 min at 56°C. The remaining steps were performed according to the protocol.

The DNA concentration was determined using the Qubit dsDNA HS Assay Kit (Thermo Fisher Scientific, Waltham, MA, USA) and a Qubit Fluorometer (Thermo Fisher Scientific). Standards were made from 10^6^ to 10^1^ copies of chromosomal DNA.

#### 2.7.3 qPCR Analysis

To design strain‐specific primers, the genome sequence of *L. gasseri* 345A was compared with other genomes in GenBank (data not shown). The primer pair Lg345A1_F (TAGTGGCATGGTGGGAGATC) and Lg345A1_R (CTACCCAAGCACCACTATCAT) were designed, and specificity and efficiency were demonstrated by analysing DNA from *L. gasseri* 345A and control *L. gasseri* strains. The primer pair did not amplify any background from control fecal samples (data not shown).

Maxima SYBR Green/ROX qPCR Master Mix (Thermo Fisher Scientific, Waltham, MA, USA) was used; primers (0.4 μM each) and 3 μL DNA were added to a total volume of 20 μL and the qPCR reactions were performed by running the program: 95°C 5 min//50×(95°C 30 s, 58°C 30 s, 72°C 30 s)//72°C 5 min, melting curve: 55°C–95°C (0.5°C/10 s). A standard curve was made by plotting the Cq values against the DNA concentrations of standard samples. Thereafter, the number of 345A cells per sample was calculated.

### 2.8 Statistics

Measurements of motility or electrophysiological data, peak fitting, event frequency detection, and non‐linear curve fitting were made using Clampfit 10 (Molecular Devices, San Jose, CA, USA). Statistics were calculated using GraphPad Prism 9.0 (GraphPad, San Diego, CA, USA). Descriptive statistics are given as means with standard deviation. In concentration‐response plots, errors are shown as standard error of the mean (SEM). The number of action potentials was summarized as medians with 25th–75th percentiles representing a discrete numeric scale. All statistics were two‐sided with a *p* < 0.05 significance level with a sample size *n*.

In the clinical study, the full analysis set (FAS) was the primary analysis consisting of all randomized subjects who had at least one dose of the investigational product and at least one post‐dose assessment of the efficacy variables. The safety set consisted of all randomized subjects receiving at least one dose of the investigational product.

Data is given as mean values with SEM. Categorical data are presented as counts and percentages. Statistical tests were carried out using an unpaired *t*‐test for comparisons between the groups or a paired *t*‐test for longitudinal observations. All statistics were two‐sided with a *p* < 0.05 significance level. Exploratory data were presented using summary statistics for observed values.

## 3 Results

### 3.1 Experimental Studies

**FIGURE 1:**
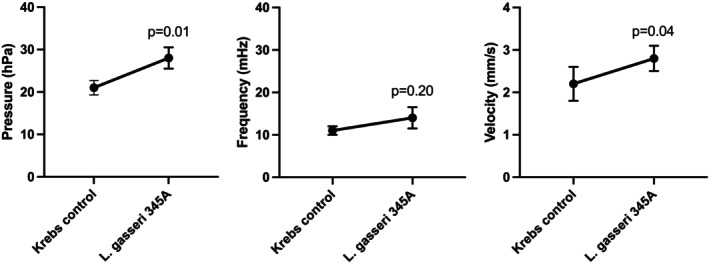
Motility‐stimulating effect intraluminal *Lactobacillus gasseri* 345A as compared with Krebs solution (control) on isolated segments from the jejunum of the mouse in vitro. Panels represent paired observations before and after adding *L. gasseri* 345A for luminal pressure, frequency, and propagation velocity of motor complexes. (*n* = 13).

Isolated segments of mouse jejunum, filled with Krebs buffer at 3 hPa input pressure, showed regular contractile activity in the form of motor complexes. The motor complexes were characterized by repeated phasic intraluminal pressure increases interspersed by periods of quiescence (data not shown). The motor complexes averaged over 20 min showed baseline amplitude 20.5 ± 2.1 hPa, frequency 11.0 ± 1.3 mHz, and velocity 2.2 ± 0.4 mm/s (*n* = 13). As shown, adding 1.4 × 10^8^ CFU of *L. gasseri* to the lumen increased the pressure amplitude by 41% (*p* = 0.01) and velocity by 31% (*p* = 0.04), but not the frequency (Figure 1).

**FIGURE 2:**
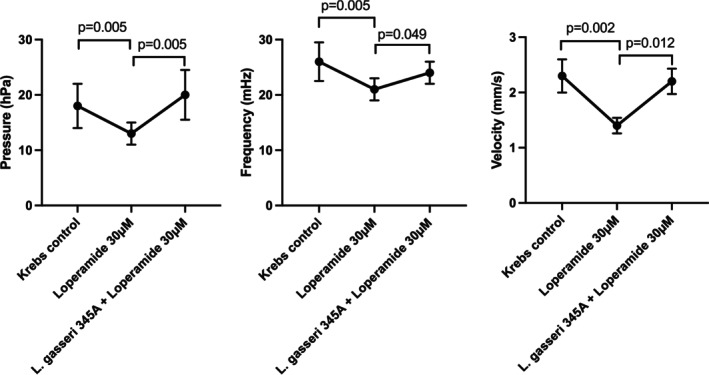
Opioid‐induced inhibition of intestinal motility by loperamide, with reversal by *Lactobacillus gasseri* 345A in vivo; *n* = 8. Left: Luminal pressure in the segment; middle: contraction frequency in the midsection of the segment; right: velocity of propagated contractions along the segment.

Using the same experimental model, the motility‐stimulating effect of *L. gasseri* bacterial suspension reversed the inhibitory effect of the μ‐opioid receptor agonist loperamide on propulsive motility (Figure 2, *n* = 8). In these experiments, *L. gasseri* 345A was added while the serosal compartment was still exposed to loperamide.

**FIGURE 3:**
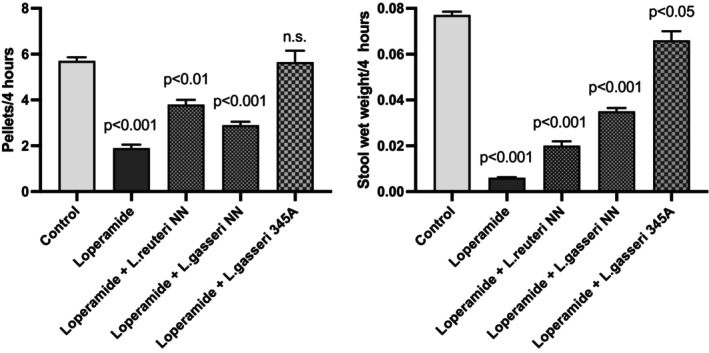
Opioid‐induced inhibition of gastrointestinal transit (left) and wet fecal weight (right) by loperamide (7–10 mg/kg) in vivo (*n* = 8). The effects of loperamide were only partially reduced by two other bacterial strains ( *Lactobacillus reuteri* NN, *Lactobacillus gasseri* NN), whereas *Lactobacillus gasseri* LG345A almost completely reversed the effect of loperamide.

The in vivo gastrointestinal transit showed that *L. gasseri* 345A given orally could counteract the action of loperamide (7–10 mg/kg; *n* = 8). As compared to controls, the *L. gasseri* strain restored the inhibitory effect of loperamide on motility (*p* < 0.001), while two other bacterial strains of *L. reuteri* and *L. gasseri* did not. Similarly, the reduction of fecal wet weight caused by loperamide (*p* < 0.001) was counteracted by *L. gasseri* but failed to completely reverse fecal weight (*p* < 0.05). The two comparative strains of *L. reuteri* and *L. gasseri* were even less effective (Figure 3).

**FIGURE 4:**
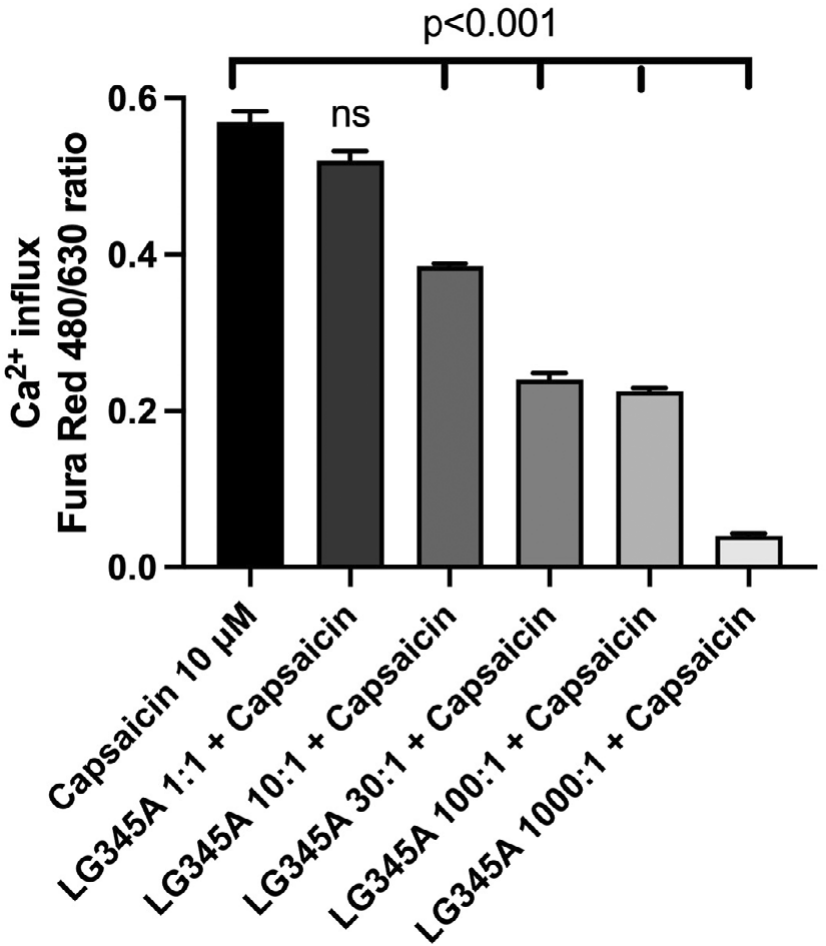
Dose‐dependent inhibition by *Lactobacillus gasseri* of capsaicin‐stimulated Ca^2+^ influx in experimental Jurkat cells. LG345A, *Lactobacillus gasseri* 345A.

Pain signaling studied on a Jurkat cell line with capsaicin‐stimulated TRPV1 ion channels showed that the medium of *L. gasseri* was able to counteract the capsaicin‐stimulated TRPV1 activity by inhibition of the Ca^2+^ influx into Jurkat cells. The full Ca^2+^ influx measured by Fura Red 488/630 ratio with 10 μM capsaicin alone (control) was dose‐dependently inhibited by increasing concentrations of *L. gasseri* 345A from dilution 1000:1 to 10:1 (Figure 4), showing that *L. gasseri* reduces the calcium influx induced by capsaicin activation of TRPV1 channels in Jurkat cells and therefore suggests that a similar mechanism may be at play in neurons expressing TRPV1.

### 3.2 Clinical Study

Sixty‐five women were screened for the study, 25 of whom discontinued before receiving any investigational product. Forty women were allocated to treatment: 20 with *L. gasseri* and 20 with placebo. Of those, 19 in the *L. gasseri* group and 18 in the placebo group were analyzed per protocol. Three subjects showed low compliance, but otherwise, there were no major protocol deviations. Demographic, medical baseline data, concomitant medication, and compliance are presented in Table 1.

**TABLE 1:** Demographics of study subjects in the full analysis set.

Item	*Lactobacillus gasseri* (*n* = 20)	Placebo (*n* = 20)
Age	39.6 ± 3.1	32.4 ± 3.0
Ethnicity	African (*n* = 1) (5%) Caucausian (*n* = 19) (95%)	American native (*n* = 1) (5%) Asian (*n* = 1) (5%) Caucasian (*n* = 18) (90%)
Body weight (kg)	67.3 ± 2.7	65.4 ± 2.1
Body height (cm)	164.4 ± 1.2	165.5 ± 1.7
BMI (kg/m^2^)	24.8 ± 0.8	23.9 ± 0.7
Concomitant medication	Contraceptives (*n* = 7)	Contraceptives (*n* = 8)
Treatment compliance	55.8 ± 0.8 of 56 capsules ingested	55.2 ± 1.2 of 56 capsules ingested
Baseline pain (VAS)	35.8 ± 6.4	21.7 ± 4.6
Baseline CSBM (no.)	0.8 ± 0.2	1.5 ± 1.1
Baseline SBM (no.)	5.0 ± 0.9	4.2 ± 0.5

Abbreviations: CSBM, complete spontaneous bowel movement; SBM, spontaneous bowel movement.

Batch analysis of the *L. gasseri* capsules showed that each capsule contained 3.6 × 10^9^ CFU, resulting in a daily dose of 7.2 × 10^9^ CFU. Despite the capsules containing more *L. gasseri* than was planned in the study protocol, there were no safety concerns observed during the study.

**FIGURE 5:**
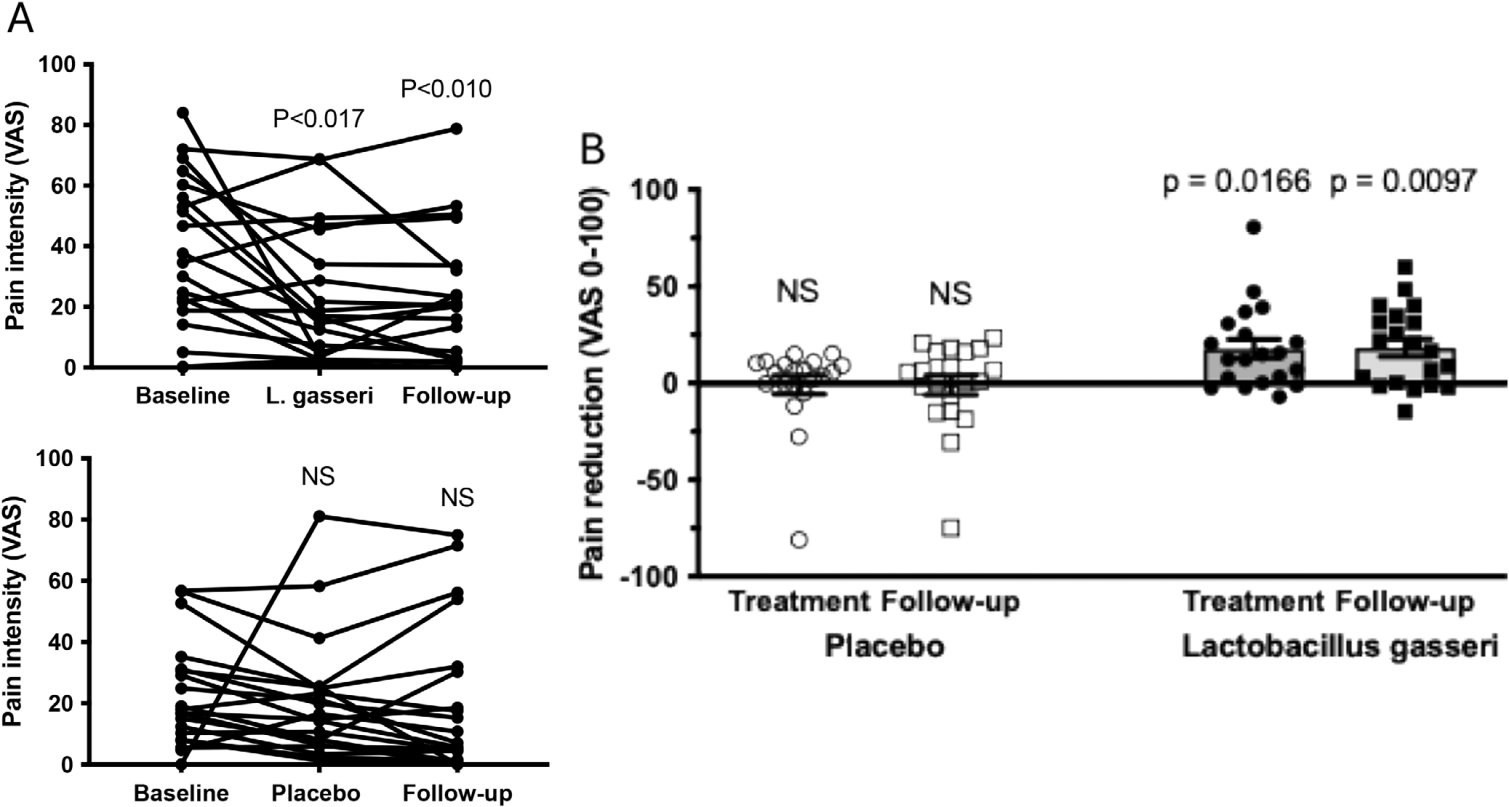
(A) Individual pain scorings at defecation in constipated patients treated with *L. gasseri* 345A (*n* = 20) as compared with placebo (*n* = 20). (B) Pain reduction from basal pain levels in constipated patients receiving *L. gasseri* 345A (*n* = 20) compared with placebo (*n* = 20). NS, non‐significant.

In the FAS analysis, the primary endpoint showed a numerical increase of CSBM by 2.8 ± 1.0 (from 0.8 ± 0.2 to 3.6 ± 1.0) in the *L. gasseri‐*treated group versus 2.0 ± 0.6 (from 1.5 ± 1.1 to 3.5 ± 0.6) with placebo (ns). As regards secondary endpoints, SBM increased from 5.0 ± 0.9 to 9.4 ± 1.6 with *L. gasseri*, while the placebo response was 4.2 ± 0.5 to 7.5 ± 0.7 (ns). Simultaneously, there was a marked reduction in pain perception on bowel movement in the *L. gasseri* ‐treated group (*p* < 0.017) as compared to placebo; an effect that was carried over into the follow‐up period (*p* < 0.010) (Figure 5). In the *L. gasseri* group, pain reduction was correlated to looser stool consistency (*r* = 0.58; *p* = 0.008), as well as to time spent for defecation (*r* = 0.68; *p* = 0.001), all different from placebo.

Among other variables investigated, we found no significant differences in terms of abdominal circumference (*L. gasseri* 83.6 ± 2.9 to 84.5 ± 2.7 cm; placebo 79.0 ± 2.0 to 78.1 ± 1.8 cm), stool consistency (*L. gasseri* 2.9 ± 0.3 to 3.5 ± 0.1; placebo stable at 3.4 ± 0.4), supplementary rescue laxatives (*L. gasseri* from 3 to 4 subjects; placebo from 3 to 1 subject) and sex life (13 abstained from sex; *L. gasseri* 52.3 ± 5.8 to 32.9 ± 6.6; placebo 37.1 ± 5.1 to 20.6 ± 4.1).

At screening, three subjects in each group used laxatives, whereas during treatment, only one in the placebo group and four in the treatment group took rescue laxatives. Three AEs were considered possibly related to treatment: two reported in the *L. gasseri* 345A group with increased abdominal circumference (moderate) and flatulence (mild) and a case of skin rash with lymphadenopathy (mild) in the placebo group. No serious AEs were reported.

At screening, *L. gasseri* 345A could not be detected by qPCR in the feces of any of the 16 and 18 subjects randomly selected from each treatment group. After the four‐week treatment period, all 16 subjects in the *L. gasseri* group had the bacterium in their feces, with 5.0 ± 0.8 log10 chromosome copies/g feces. None of the placebo‐treated subjects had *L. gasseri* in their feces. At follow‐up, one of the tested subjects in the *L. gasseri* group had 4.6 log10 chromosome copies/g of the bacterium in the feces, whereas none of the subjects were in the placebo group.

## 4 Discussion

Similar to the findings of our present study, *Lactobacillus gasseri* strains have shown effects in relation to pain, gastrointestinal motility, and constipation. In a study by Kim et al. [18], intake of the strain *L. gasseri* BNR17 in patients with irritable bowel syndrome (IBS) the abdominal pain score Focusing on the possible mechanism of action shows that *L. gasseri* strongly adheres to the intestinal epithelial cell lining in order to restore and reinforce the epithelial barrier, as shown in an in vitro model comprising Caco‐2 cell monolayers sensitized with hydrogen peroxide [19]. Furthermore, in a rat model of IBS, *L. gasseri* LA806 significantly reduced visceral hypersensitivity induced by chronic stress, as shown in a workshop by the same research group (*Ann Nutr Metab* 2019;74:14). In extension to this, Olivares et al. [20] showed that intake of a probiotic yogurt containing *L. gasseri* resulted in significantly higher fecal volume, water content, and higher stool frequency as compared to controls.

We studied a new strain of *L. gasseri* 345A in vitro and in vivo, using motility recordings and experimental studies on cellular Ca^2+^ influx to substantiate findings for a clinical study on constipation in women. In our preclinical studies, we found significant stimulatory effects of *L. gasseri* (345A) on propulsive motility of the small bowel. There is a caveat to this finding since effects on chronic constipation are usually known to be linked to colonic motility alterations. However, data indicate that jejunal motility is also affected in chronic constipation [12, 13] why stimulation of jejunal motility and propulsion should be of relevance for the delivery of luminal contents into the colon and enhance transit. Our results showed significant inhibitory effects of *L. gasseri* 345A on Ca^2+^ fluxes over cellular membranes induced by capsaicin in Jurkat cells. This capsaicin‐sensitive mechanism was shown to be mediated by the TRPV1, known as the vanilloid receptor 1, encoded by the *TRPV1* gene in humans [21, 22]. TRPV1 is a mechanosensitive ion channel that regulates body temperature and mediates nociceptive pain through primary afferent sensory neurons. TRPV1 also cooperates with the transient receptor potential ankyrin 1 (TRPA1) to detect noxious stimuli [23, 24]. Against this background, we carried out a clinical exploratory study in constipated women being a patient category for whom we considered it possible to achieve meaningful results with *L. gasseri* treatment. Our study showed only a tendency for an increased number of CSBMs and SBMs under treatment with *L. gasseri* as compared to placebo. Instead, we found a pain‐relieving effect of *L. gasseri* , which should fit with our preclinical data showing bacterial inhibition of the capsaicin‐induced Ca^2+^ influx in a Jurkat cell line. At the same time, the pain‐relieving effect was related to the bowel movements and associated with stool consistency and time spent for defecation.

Probiotics have shown promising results for the treatment of constipation in both animals and humans [21, 22, 23, 24]. Nevertheless, the mechanism of action of *L. gasseri* on gut motility and constipation is yet unclear. There is one systematic review and meta‐analysis showing that specific probiotics at doses from 3 × 10^10^ to 1 × 10^8^ CFU per day decrease gastrointestinal transit time by 12 h and increase stool frequency by 1.5 stools per week, along with symptom improvement [25].

The enteric nervous system functions independently from the CNS with intramural pathways for motor and sensory function [26, 27, 28]. Studies in germ‐free mice show that bacterial colonization of the gut is important for the development and maturation of enteric nerves [29, 30]. Metabolic products from intestinal fermentation, such as short‐chain fatty acids and peptides, can stimulate the enteric nervous system and promote gastrointestinal transit [29]. Neuroendocrine cells in the gut can also interact with microbiota [31] via serotonin (5‐HT) [32]. 5‐HT is produced in both the enteric and central nervous systems as a key neurotransmitter with a pivotal role for motor and secretory reflexes in the enteric nervous system [33]. Hence, the gut microbiota plays a crucial role in the development of the enteric nervous system [34] and can interact with the central nervous system (CNS) and gastrointestinal tract [35] through a “microbiota‐gut‐brain axis” [36, 37, 38].

The bacterium *Limosilactobacillus reuteri* DSM 17938 has been shown to modulate neuronal‐dependent motility reflexes that communicate with the brain in the mouse [39]. In the rat, *Lactobacillus reuteri* interacts with the gut‐brain axis through the modulation of afferent sensory neurons that influence gut motility [40]. Some probiotic species can affect brain activity in humans [41], but effects on gut motility via central nervous modulation are yet unclear [42]. *L. reuteri* has been shown to increase the excitability of myenteric neurons in rats, and supernatants from the *Escherichia coli* strain Nissle increase maximal tension forces of smooth muscle from the human colon in vitro [43]. Thus, the microbiome can operate as a modulator of visceral pain [37].

Our exploratory study suggests that the bacterial microbiota is involved in the sensory mechanisms related to bowel emptying. Patients committed to active treatment with *L. gasseri* 345A experienced bowel emptying with less pain; the alleviation of which, with looser stool and less time spent for defecation, indicates greater satisfaction with their bowel habits [44]. Since the effect was carried over into the follow‐up period, we cannot exclude the possibility of a placebo response in keeping with the four‐week treatment effect since the bacterium is not expected to colonize the colon for prolonged periods [45]. Hence, to achieve a therapeutic goal, the bacterium should be administered on a long‐term basis.

The pain‐relieving effect in humans is supported by animal experimentation [46]. Perez‐Burgos et al. [47] show that a bacterium of the same family, *Limosilactobacillus reuteri* DSM 17938, acts on TRPV1 channels as a target for an antinociceptive effect. In line with this, our experiments using *L. gasseri* showed a similar effect on TRPV1 channels, suggesting a biological effect on pain sensations.

Even though our findings can be ascribed to a shift of the intestinal microbiota, our study is limited by the small number of individuals studied. As shown, the variable responsiveness among constipated subjects is considerable. A larger sample would be expected to reduce this variability. Since the enrollment of subjects took place with a simplistic inclusion as described by the Rome III criteria and exclusion of confounding diseases, we may have overlooked other determinants in our study. Since our study showed the most prominent effect of *L. gasseri* 345A on pain, a specific evaluation of constipation with a strong pain component should preferably be studied. Similarly, data indicative of increased bowel movements with *L. gasseri* 345A suggest that select patients with a stronger constipation profile should be studied. However, this would diminish the applicability of the study as many with less outspoken constipation express severe symptoms.

## Author Contributions

S.R. study planning and microbiology, A.‐L.D. clinical studies, first draft, results summary and conclusions, Y.‐K.M. preclinical motility studies, A.P. molecular microbiology, A.M.S. preclinical motility and neurophysiology, P.F. preclinical cell physiology, W.K. preclinical motility studies, P.M.H. clinical trial, first motility results and conclusions.

## Conflicts of Interest Statement

The authors declare no conflicts of interest. Author S.R. discloses part‐time employment at BioGaia AB.

## Data Availability

The data that support the findings of this study are available from the corresponding author upon reasonable request.
